# Artificial intelligence in electrocardiogram-based prediction of heart failure: a systematic review and meta-analysis

**DOI:** 10.3389/fcvm.2025.1659298

**Published:** 2026-01-02

**Authors:** Shunhong Zhang, Jun Jiang, Yi Luo, Guangyue Liu, Saidi Hu, Siran Wan, Chenchen Luo, Hong Li, Nian Li, LinYong Zhao

**Affiliations:** 1 Department of Cardiology, Pangang Group General Hospital, Panzhihua, China; 2 The 19th Batch of Chinese Medical Team in Sao Tome and Principe, Sichuan Provincial Health Commission, West China Hospital, Sichuan University, Chengdu, China; 3 Department of Anesthesiology, West China Hospital, Sichuan University, Chengdu, China; 4 Department of Stomatology, Yaan people’s Hospital, Yaan, China; 5 Department of Gynaecology and Obstetrics, Yaan people’s Hospital, Yaan, China; 6 Department of Outpatient Chengbei, The Affiliated Stomatological Hospital, Southwest Medical University, Luzhou, China; 7 Department of Ultrasound Medicine, Panzhihua Women & Enfants Healthcare Hospital, Panzhihua, China; 8 Department of Traditional Chinese Medicine, Panzhihua Central Hospital, Panzhihua, China; 9 Department of General Surgery & Laboratory of Gastric Cancer, State Key Laboratory of Biotherapy/Collaborative Innovation Center of Biotherapy and Cancer Center, West China Hospital, Sichuan University, Chengdu, China; 10 Gastric Cancer Center, West China Hospital, Sichuan University, Chengdu, China

**Keywords:** artificial intelligence, electrocardiogram (ECG), heart failure, deep learning, predictive modelling, meta-analysis

## Abstract

**Background:**

Heart failure (HF) continues to pose a significant global health challenge, characterized by an increasing prevalence. Early identification of individuals at the highest risk of developing HF and implementing interventions can prevent and delay disease progression. The application of artificial intelligence (AI) to electrocardiograms (ECGs) presents a novel strategy for early prediction; however, the effectiveness and generalizability of this approach necessitate systematic evaluation.

**Objective:**

To systematically evaluate the performance of AI models based on ECGS in predicting HF.

**Methods:**

This study was registered on PROSPERO (CRD420251012231). Following the PRISMA guidelines, we conducted a systematic literature search across multiple databases, including PubMed, IEEE Xplore, Medline, and Embase, for studies published between 2005 and 2025. The inclusion criteria focused on AI models based on ECGs that reported performance metrics such as the AUROC (Area Under the Receiver Operating Characteristic Curve)/C-statistic. Meta-analysis was performed by employing a random-effects model to evaluate the efficacy of AI in predicting HF through the pooled AUROC/ C-statistic. Additionally, we conducted heterogeneity analyses using I^2^ and performed subgroup comparisons across various ethnicities, while assessing the risk of bias with the PROBAST + AI tool.

**Results:**

A total of five studies involving 11 cohorts and 1,728,134 participants were included in the analysis. The pooled AUROC/C-statistic was found to be 0.76 (95% CI: 0.74–0.78; *p* < 0.001), indicating moderate-to-good discrimination capability. Subgroup analyses demonstrated consistent performance across different ethnic groups, with AUROC values ranging from 0.77 to 0.79, comparable to the traditional model which had an AUROC of 0.742 (95% CI: 0.692–0.787, *P* = 0.575). Notably, significant heterogeneity was observed among the studies (*I*^2^ = 89%, *p* < 0.01), which may be attributed to systematic differences in population characteristics, study design, and data quality.

**Conclusions:**

Theoretically, artificial intelligence-enabled electrocardiogram (AI-ECG) models demonstrate promising applicability for predicting HF; however, their effectiveness remains uncertain due to a high risk of bias and a lack of clinical validity studies.

**Systematic Review Registration:**

https://www.crd.york.ac.uk/PROSPERO/view/CRD420251012231, PROSPERO CRD420251012231.

## 1 Introduction

HF affects over 64 million people globally, with its incidence and prevalence continuing to rise (1). Patients with HF experience a decline in quality of life, an increased risk of mortality, and a significant economic burden (2, 3). Notably, identifying individuals at high risk for future HF can mitigate these risks through the early initiation of low-cost medical treatments, which has been demonstrated to be able to alter the disease trajectory in clinical practice guidelines, reduce the risk of new-onset clinical HF, and improve life expectancy (4, 5). Although strategies based on serum testing (6, 7) and clinical scoring (8, 9) to predict new-onset HF are feasible, their reliance on invasive blood sampling and the complexity of acquiring multivariable parameters substantially increase implementation challenges and associated costs. In stark contrast, AI-ECG analysis, which detects latent cardiovascular disease features via 12-lead ECGs, provides a non-invasive and cost-effective solution.

Pioneering work in the deep ECG phenotyping field has demonstrated that AI can effectively predict atrial fibrillation (10, 11). In recent years, research has also shown that AI-ECG exhibits good performance in predicting HF (12–14); however, to our knowledge, despite continuous technological advancements, a comprehensive systematic evaluation of its performance for HF prediction is still lacking. Therefore, this study aims to assess its discriminatory ability and heterogeneity in prediction through a meta-analysis, providing clinicians with a more comprehensive understanding of the application of AI-ECG in HF prediction, thereby guiding future clinical practices and research directions.

## 2 Methods

### 2.1 Search strategy and inclusion criteria

This review adheres to the PRISMA statement and CHecklist for critical Appraisal and data extraction for systematic Reviews of prediction Modelling Studies (CHARMS) (15, 16) (Supplementary Table 1). Furthermore, this study has been registered with PROSPERO (CRD420251012231). A systematic search of the literature published from March 2005 to March 2025 was conducted in the PubMed, IEEE Xplore, Medline, and Embase databases. The search terms used were (“Artificial Intelligence” OR “Machine Learning” OR “Deep Learning”) AND (“Electrocardiogram” OR “ECG”) AND (“Heart Failure” OR “congestive heart failure” OR “cardiac insufficiency” OR “Cardiac Failure”) AND (“Prediction” OR “Early Detection”). The inclusion criteria were: (1) Artificial intelligence models developed solely based on 12-lead ECG data for the prediction of heart failure; (2) Reporting of discriminative performance metrics (such as AUROC, c-statistic) and 95% confidence intervals (CIs); (3) Original research published in English and subjected to peer review. The exclusion criteria encompassed commentary, Conference Paper, reviews, non-English literature, models integrating non-ECG variables (such as laboratory indicators), and studies that did not provide complete performance metrics.

### 2.2 Data extraction, assessment of quality and risk of bias

Two independent reviewers extracted data using a standardized form that captured study characteristics, including author, year, cohort, and sample size, as well as the AI model architecture and performance metrics. Discrepancies were resolved through consensus or third-party adjudication. The risk of bias and quality were assessed using the PROBAST + AI tool, which evaluates domains such as participants and data sources, predictors, outcomes, and analysis methodology (17). Studies were classified as having low, unclear, or high risk of bias based on predefined criteria. Cochran's *Q* test and the *I*^2^statistic were employed to quantify heterogeneity. A sensitivity analysis was conducted by sequentially removing individual studies based on a random effects model.

### 2.3 Data synthesis and statistical analysis

In a single study, we evaluated the c-statistic/AUROC of the model. When a study reported multiple cohorts and presented data for each cohort separately, we evaluated the model performance of each cohort in the study individually. Funnel plots were created to examine publication bias. We analyzed the discriminative ability through summary measures of the AUROC/c-statistic and the corresponding 95% CI. When the 95% confidence interval is not reported, we calculated it using the method described by Debray et al. (18). We calculated the 95% prediction interval (PI) to describe the degree of inter-study heterogeneity and indicate the possible range of the predicted model performance in new validations. Based on previous literature, summary AUROC/c-statistics were predefined as inadequate (<0.60), sufficient (0.60–0.70), acceptable (0.70–0.80), and excellent (>0.80) (19). We performed a meta-analysis using the metafor package in R (R Foundation for Statistical Computing 4.5.0). Our primary analysis evaluated the overall discriminative ability of all models for which cohorts reported AUROC/c-statistic data. In the secondary analysis, we compared the AI-ECG model with traditional FHS-HF/PCP-HF evaluation models. One researcher rated the certainty of the evidence for the primary results, while another researcher conducted a review.

## 3 Results

### 3.1 Study selection

**Figure 1:**
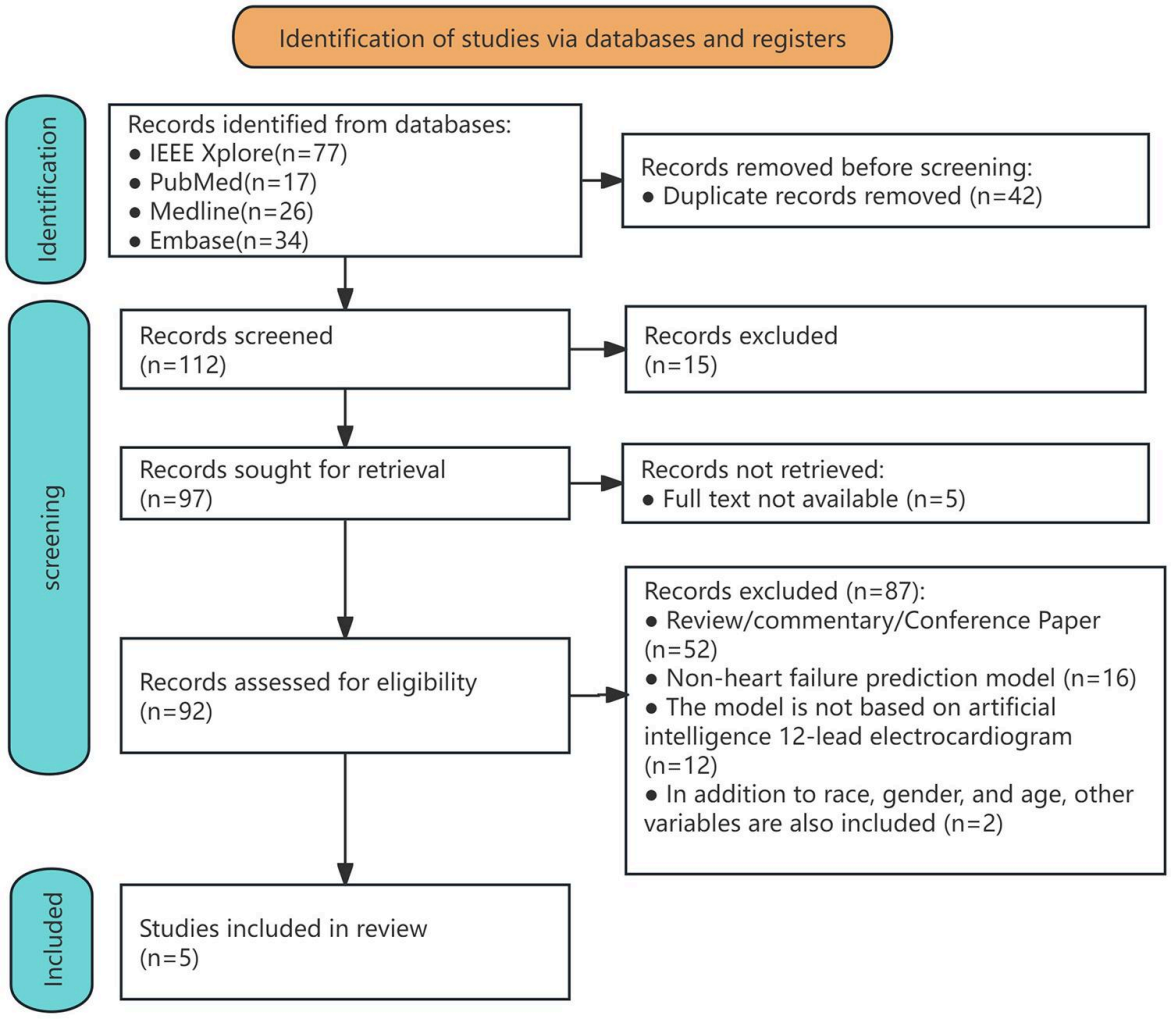
Flowchart of the process for including studies in the systematic review and meta-analysis.

A systematic literature search conducted across PubMed, IEEE Xplore, Medline, and Embase identified 154 initial records (Supplementary Table 2). After the removal of duplicates, 112 unique studies underwent title and abstract screening. Of these, 20 articles were excluded due to irrelevance and unavailability of full texts. The remaining 92 full-text articles were assessed for eligibility. A total of 87 studies were excluded for the following reasons: 52 were classified as reviews, commentaries, or conference papers; 16 studies did not integrate HF prediction models; 12 prediction models were not based on artificial intelligence or 12-lead ECGs; and two studies included non-ECG variables alongside ethnicity, gender, and age. Ultimately, five studies met all inclusion criteria and were included in the systematic review and meta-analysis (Figure 1). Applying the PICOTS framework to clarify the intended objectives or purposes of predictive model evaluation (Supplementary Table 3).

### 3.2 Study characteristics

The analysis included five studies comprising 11 independent cohorts, totaling a sample size of 1,728,134 participants. The cohorts were predominantly from the United States (7 out of 11), with additional representation from Asia (1 out of 11, TSGH), Europe (1 out of 11, UKB), and South America (1 out of 11, ELSA-Brasil in Brazil). Sample sizes varied significantly across the cohorts, ranging from 6,736 participants in the MESA cohort to 539,934 in the TSGH cohort. The mean age of participants ranged from 51 years to 62.2 years, with female representation varying between 49.6% and 56.6%. Follow-up durations varied from 3.1 months to 6.8 years (Table 1).

**Table 1:** Characteristics of included studies.

Study	Cohort(country)	HF risk prediction method	Ethnicity	HF cases/total patients (%)	Age (years, mean ± SD)	Female sex (%)	Outcome	Outcome coding	Enrolment period (mean f/u in years)	Exclusion criteria
Kaur et al. (2024) (20)	SUMC(USA)	Deep learning	Overall (undifferentiated)	59,816/326,663 (18.3%)	59.3 (−)	162,190 (49.6%)	HF	SNOMED	2008–2018 (6.8)	Lack of follow-up or prior HF
			Asian	8,622/46,393 (18.6%)	N/S	N/S	HF	SNOMED	2008–2018 (6.8)	Lack of follow-up or prior HF
			Hispanic	7,042/40,301 (17.5%)	N/S	N/S	HF	SNOMED	2008–2018 (6.8)	Lack of follow-up or prior HF
			Non-Hispanic White	35,086/184,410 (19.0%)	N/S	N/S	HF	SNOMED	2008–2018 (6.8)	Lack of follow-up or prior HF
			Black or African American	3,066/13,063 (23.5%)	N/S	N/S	HF	SNOMED	2008–2018 (6.8)	Lack of follow-up or prior HF
Akbilgic et al. (2021) (12)	ARIC (USA)	Deep learning	Undifferentiated	803/14,613 (5.5%)	54(± 5)	7,978 (55%)	HF	ICD-9-CM	1987-1989 (−)	Individuals with HF, those with missing HF data during follow-up, and those with missing or poor-quality ECGs
Dhingra et al. (2025) (13)	YNHHS (USA)	Deep learning	Undifferentiated	9,645/231,285 (4.2%)	57(-)	130,941 (56.6%)	HF/LVEF < 50%	ICD-10-CM	2014–2023 (4.5)	Prior HF diagnostic codes, abnormal echocardiogram findings (LVEF < 50% or severe diastolic dysfunction), and hospitalization for HF within 3 months after baseline ECG examination
	UKB(UK)	Deep learning	Undifferentiated	46/42,141 (0.1%)	65 (−)	21,795 (51.7%)	HF	ICD-10-CM	2014–2021 (3.1)	Hospitalization records before baseline ECG examination showed diagnostic codes for HF
	ELSA-Brasil(BRA)	Deep learning	Undifferentiated	31/13,454 (0.2%)	51 (−)	7,348 (54.6%)	HF	C-EEMRR	N/S(4.2)	With pre-existing HF or baseline echocardiogram showing LVEF < 50%
Lin et al. (2025) (14)	TSGH (CHN)	Deep learning	Asian	(–)/1,078,629	60.2(± 14.5)	705,153 (49.8%)	HF	ICD-9/10-CM	N/S(5)	Under 30 years old; with pacemakers; without medical records in the system within one year after ECG; with a history of HF, MI, or IS; ECGs with acquisition dates matching the date of diagnosis
Butler et al. (2023) (21)	ARIC (USA)	Deep learning	Undifferentiated	803/14,613 (5.5%)	54(± 5)	7,978 (55%)	HF	ICD-9-CM	1987–1989(N/S)	Baseline HF; missing ECGs
	MESA (USA)	Deep learning	Undifferentiated	239/6,736 (3.5%)	62.2(±10.2)	3,555 (52.8)	HF	ICD-9/10-CM or symptoms	N/S	Missing baseline ECGs

AF, atrial fibrillation; SUMC, Stanford University Medical Center; ARIC, atherosclerosis risk in communities; YNHHS, Yale New Haven Health System; UKB, UK Biobank; ELSA-Brasil, Brazilian longitudinal study of adult health; TSGH, tri-service general hospital; MESA, multi-ethnic study of atherosclerosis; ECG, electrocardiogram; LVEF, left ventricular ejection fraction; SNOMED, systematized nomenclature of medicine; C-EEMRR, clinical events based on expert medical record review; N/S, not specified.

**Table 2:** Methodological characteristics of AI-ECG models in included studies.

Study	Model Type	Input Format	Preprocessing Steps	Validation Strategy
Kaur et al. (2024) (20	CNN-based	Raw waveform	Filtering, resampling	Internal validation (ethnicity subgroups)
Akbilgic et al. (2021) (12)	CNN-LSTM hybrid	Raw waveform	Noise removal, amplitude normalization	Train-validation split
Dhingra et al. (2025) (13)	CNN (image-based)	ECG image	Image standardization, lead extraction	External validation (UKB, ELSA)
Lin et al. (2025) (14)	Multitask DL	Raw waveform	Bandpass filtering, segmentation	Temporal validation
Butler et al. (2023) (21)	CNN-based	Raw waveform	Baseline wander removal, resampling	Internal & external (MESA)

CNN, convolutional neural network; LSTM, long short-term memory; UKB, UK Biobank; ELSA-Brasil, Brazilian longitudinal study of adult health; MESA, multi-ethnic study of atherosclerosis; ECG, electrocardiogram.

All studies employed deep learning as the core model architecture. Two studies (12, 21) utilized the same ARIC cohort (*n* = 14,613) but reported distinct analyses. The outcome definitions varied; most studies relied on clinical HF diagnoses using ICD codes, while the YNHHS cohort (13) incorporated echocardiographic criteria, specifically left ventricular ejection fraction (LVEF) less than 50%. Exclusion criteria predominantly focused on pre-existing HF, missing ECGs, or incomplete follow-up data. Notably, none of the included studies evaluated clinical utility or cost-effectiveness, and all lacked external validation in geographically diverse settings. The largest cohort (14) derived data from electronic health records, which may introduce unmeasured confounders, whereas smaller cohorts, such as MESA, emphasized community-based populations with detailed phenotyping.

### 3.3 AI model methodological characteristics

Although all included studies employed deep learning architectures, there was considerable diversity in model design, input representation, and validation strategies, which may influence performance and generalizability (Table 2). Input formats varied between raw signal time-series and image-based ECG representations, which may affect feature extraction robustness. Preprocessing also differed, though most studies applied noise filtering and signal normalization. Only two studies (13, 21) included external validation cohorts, highlighting a general limitation in geographic and clinical generalizability.

### 3.4 Risk of bias assessment

**Figure 2:**
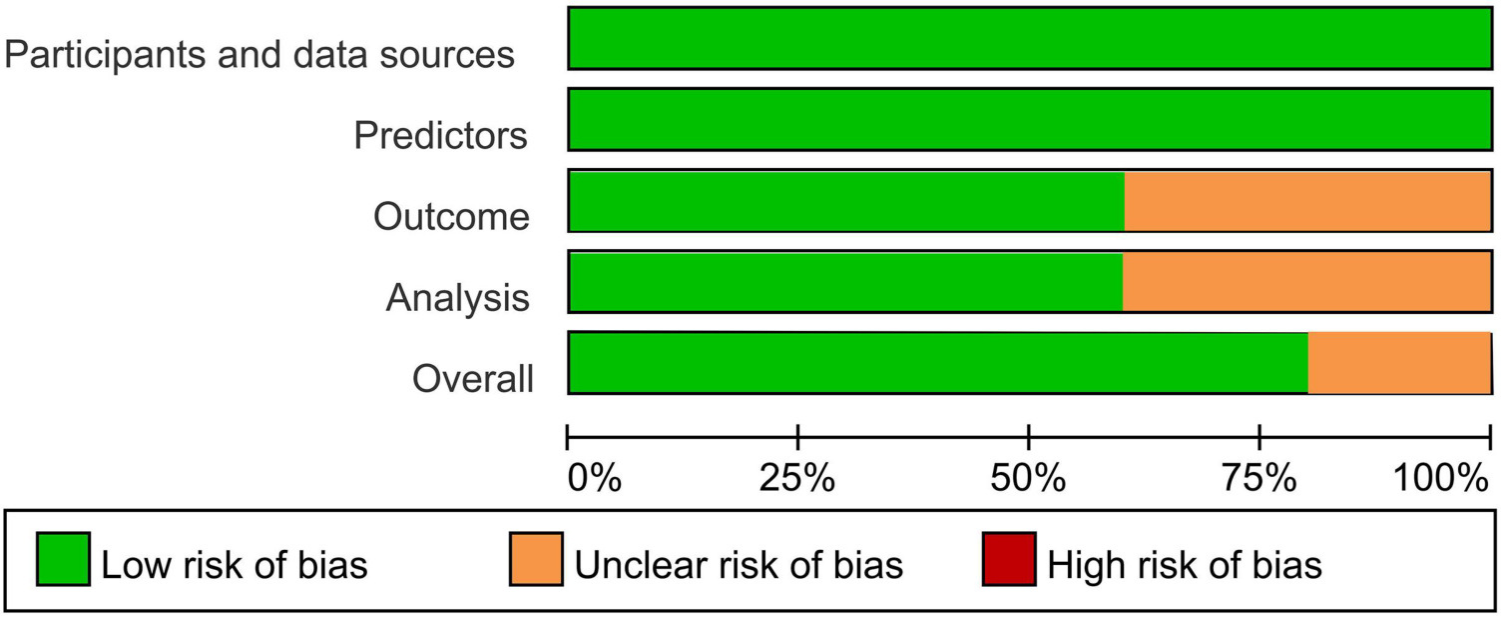
Risk of bias across all included studies.

Most AI-ECG models (80%) demonstrate a low overall risk of bias. The unclear risk primarily stems from ambiguous reporting of result intervals and a lack of procedures for handling missing data (Figure 2; Supplementary Table 4).

### 3.5 Heterogeneity and sensitivity analysis

**Figure 3:**
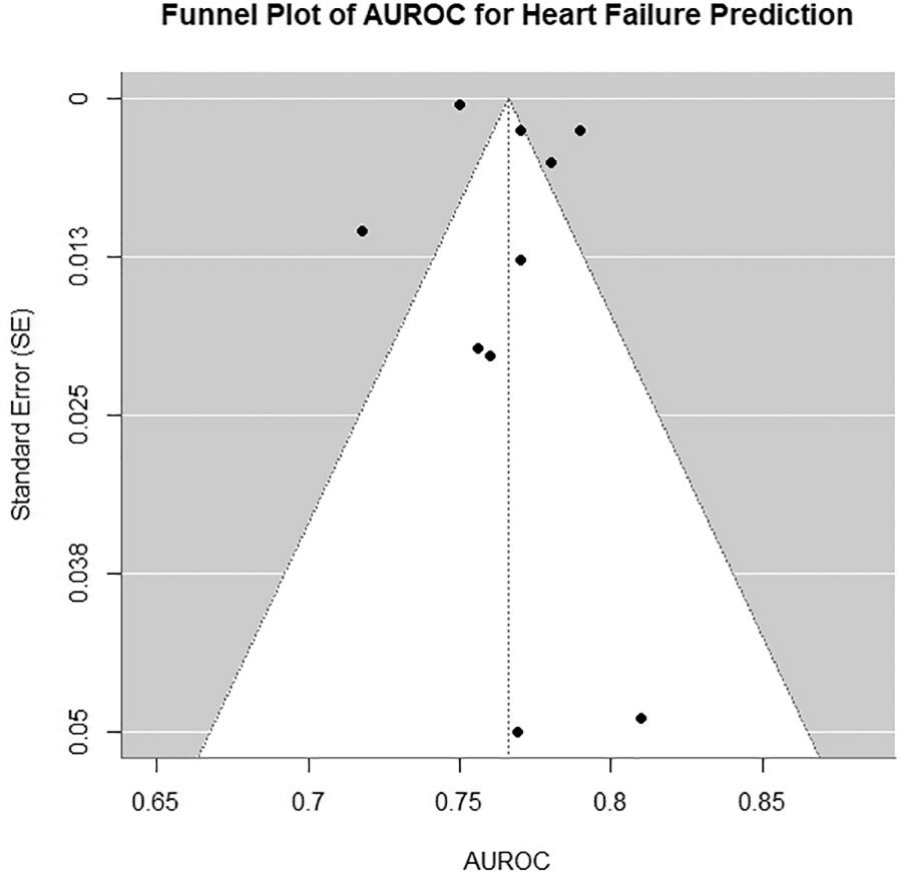
Funnel plot of all models included in primary meta-analysis. AUROC, area under the receiver operating characteristic curve.

Significant heterogeneity was observed across studies (*I*^2^ = 89%, Cochran's Q = 98.7, *p* < 0.001). Funnel plots were symmetrical but exhibited additional horizontal scatter (Figures 3), which is consistent with the presence of between-study heterogeneity. This heterogeneity was driven by three primary factors: (1) variability in outcome definitions (e.g., clinical HF vs. HF with LVEF < 50%); (2) diversity in data sources (hospital-based vs. community cohorts with differing baseline risk profiles); and (3) methodological inconsistencies in ECG preprocessing and model hyperparameter optimization. To assess the robustness of the pooled AUROC [0.76 (0.74–0.78)], we conducted a leave-one-out sensitivity analysis. After excluding any single study, the pooled estimates showed minimal changes (range: 0.75–0.77). When the Dhingra (13) (ELSA-Brasil) cohort with the highest AUROC was excluded, the pooled estimate slightly decreased to 0.75 [0.73–0.77]. Upon excluding the UKB cohort with the lowest precision, the confidence interval narrowed to 0.76 [0.74–0.78]. The sensitivity analysis confirmed the robustness of the pooled results.

### 3.6 Heart failure outcome definition

The definition of HF, the primary outcome, varied across the included studies. Most studies defined HF based on clinical diagnoses recorded in hospital administrative databases using ICD codes. In contrast, one study (13), YNHHS cohort) applied a more stringent, imaging-based criterion, defining HF as a LVEF of less than 50%. This methodological distinction between broadly defined, clinically coded HF and a more specific, phenotypically defined HF was identified *a priori* as a major potential source of clinical heterogeneity.

### 3.7 Model performance

**Figure 4:**
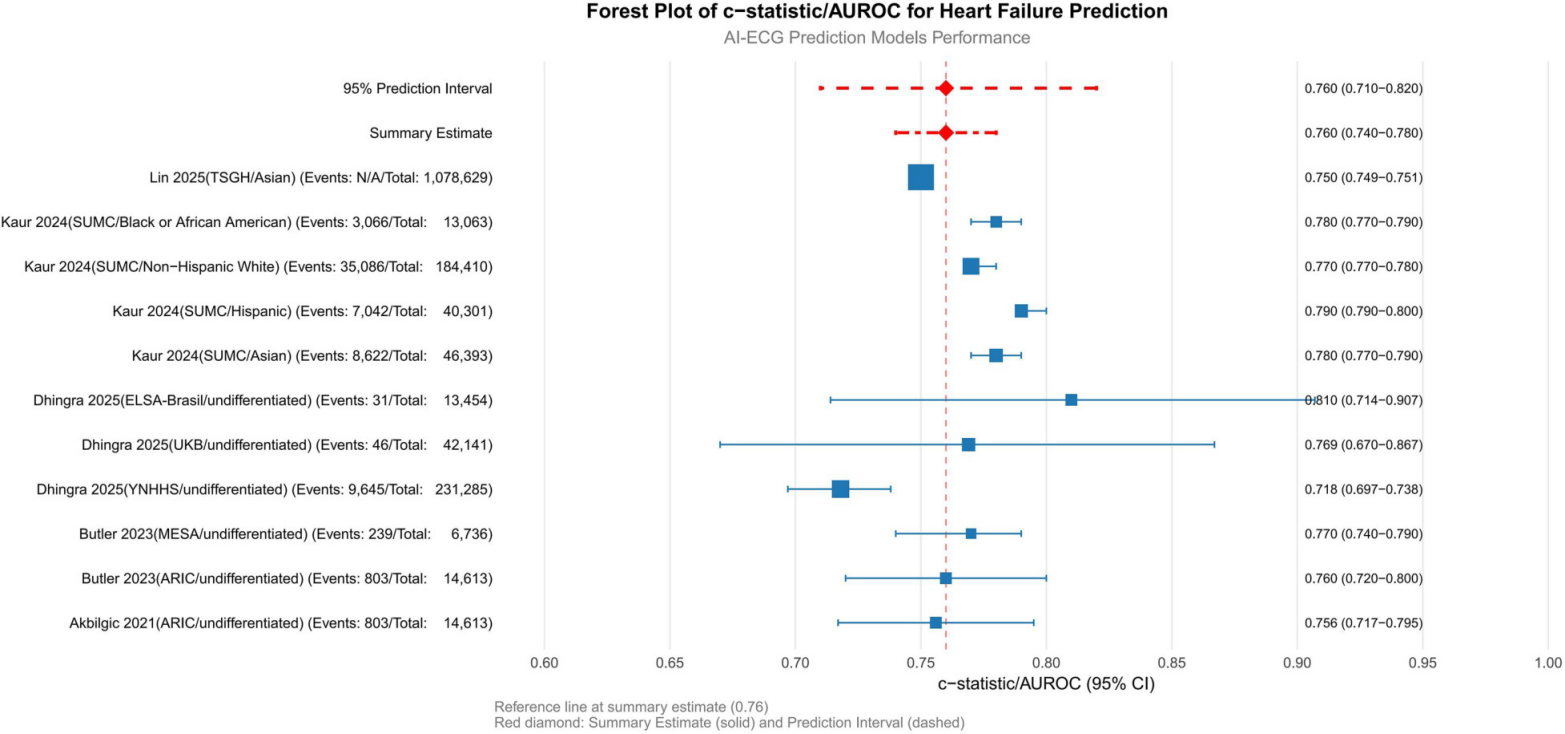
Primary analysis: meta-analysis of c-statistics/AUROC. SUMC, Stanford University Medical Center; ARIC, atherosclerosis risk in communities; YNHHS, Yale New Haven health system; UKB, UK Biobank; ELSA-Brasil, Brazilian longitudinal study of adult health; TSGH, tri-service general hospital; MESA, multi-ethnic study of atherosclerosis; AUROC, area under the receiver operating characteristic curve.

The pooled AUROC across all cohorts was 0.76 (95% CI: 0.74–0.78; *p* < 0.001), indicating moderate-to-good discriminatory ability for HF prediction (Figure 4). Subgroup analyses revealed nuanced variations: among ethnic subgroups, the highest AUROC was observed in Asian populations [0.78 (0.77–0.79), (20)], while Hispanic and non-Hispanic White cohorts showed comparable performance [0.79 (0.79–0.80) and 0.77 (0.77–0.78), respectively], suggesting minimal ethnic disparity. Undifferentiated cohorts (e.g., ARIC and MESA) exhibited consistent AUROCs (0.756–0.77) despite differences in sample size and HF prevalence (3.5%–5.5%). Geographic variability highlighted challenges in low-incidence settings [e.g., UKB: AUROC 0.769 (0.670–0.867), HF incidence 0.1%], whereas smaller cohorts with limited events (e.g., ELSA-Brasil: *n* = 31) achieved higher point estimates [AUROC 0.810 (0.714–0.907)] but with wider confidence intervals, underscoring the impact of sample size and event frequency on precision.

### 3.8 Comparison with traditional models

**Figure 5:**
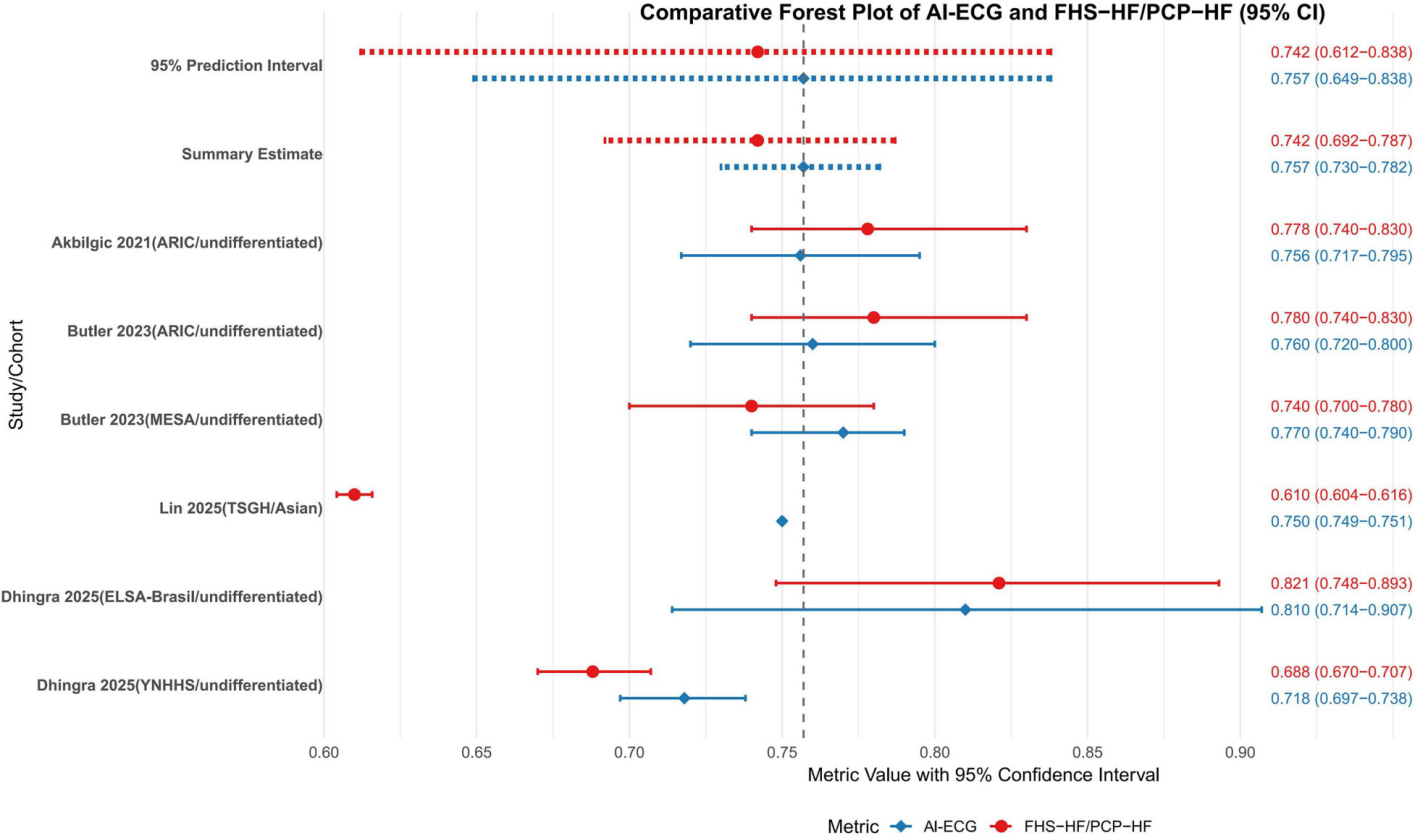
Meta-analysis of AI-ECG model and FHS-HF/PCP-HFC model c-statistics/AUROC. SUMC, Stanford University Medical Center; ARIC, atherosclerosis risk in communities; MESA, multi-ethnic study of atherosclerosis; ELSA-Brasil, Brazilian longitudinal study of adult health; AUROC, area under the receiver operating characteristic curve; PCP-HF, pooled cohort equations to prevent HF; FHS, Framingham heart study; AI-ECG, artificial intelligence electrocardiogram.

The head-to-head comparison between AI-ECG and traditional FHS-HF/PCP-HF risk scores across 5 cohorts from 3 studies demonstrated a pooled AUROC of 0.757 (95% CI: 0.730–0.782) for AI-ECG models vs. 0.742 (95% CI: 0.692–0.787) for traditional models, with overlapping confidence intervals and a non-significant test for subgroup differences (*P* = 0.575) indicating no consistent statistical superiority for the AI-ECG approach (Figure 5). Despite this overall equivalence, population-specific advantages emerged, as the AI-ECG model showed significantly superior performance in the large Asian TSGH cohort (AUROC 0.75 vs. 0.61, *P* < 0.001) and numerically higher discrimination in the MESA cohort, whereas performance remained comparable in the ARIC and other SUMC sub-cohorts. The substantial 95% prediction intervals for both AI-ECG (0.649–0.838) and traditional models (0.612–0.838) reflect considerable uncertainty in their relative performance in new settings. From a clinical perspective, the observed absolute AUROC difference of approximately 0.015, while not statistically significant at the overall level, may still hold relevance in population-wide screening contexts where even modest improvements in discrimination can meaningfully reclassify risk categories for a substantial number of individuals, potentially enabling more targeted preventive interventions.

### 3.9 Subgroup analysis by heart failure definition

**Figure 6:**
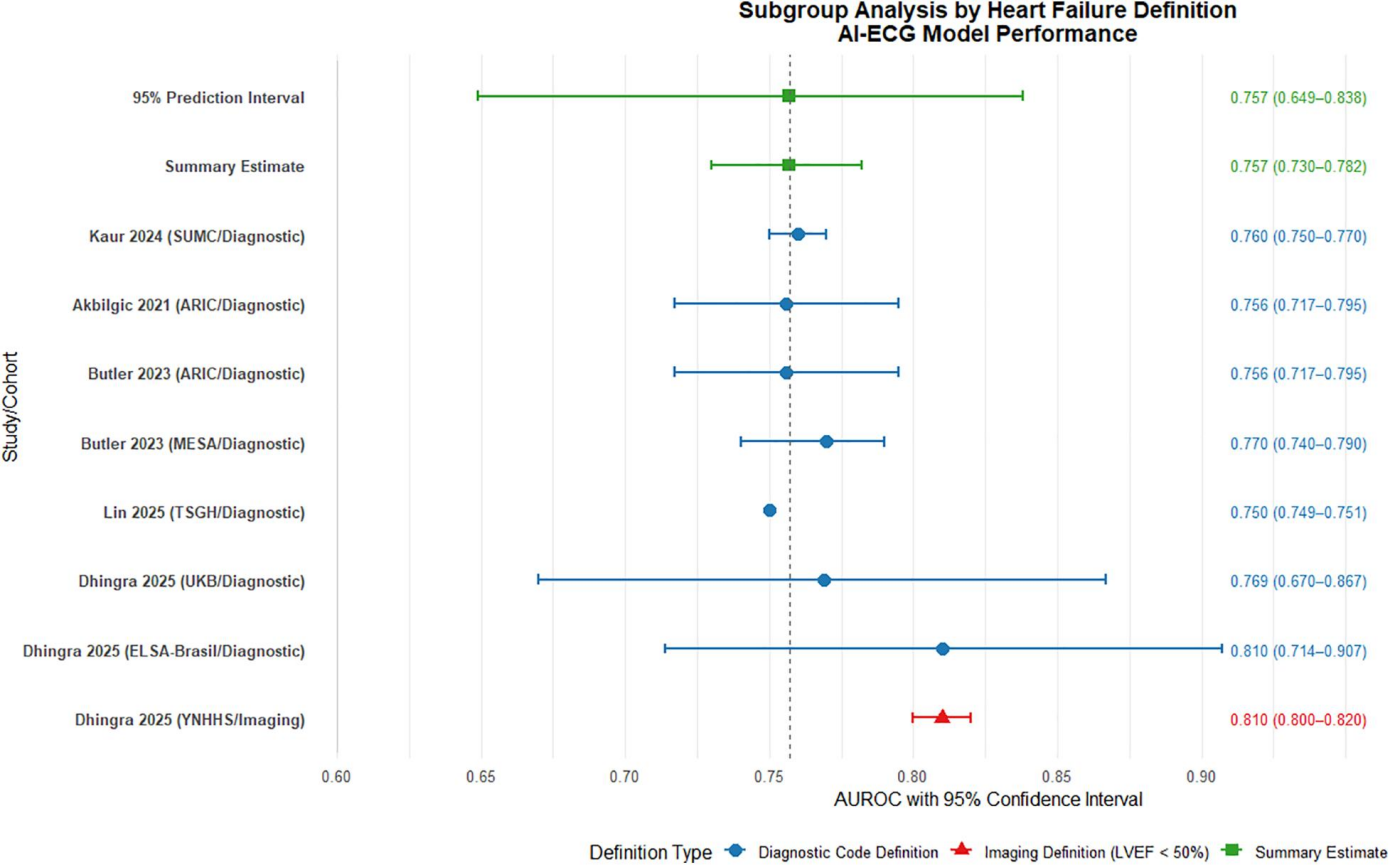
Subgroup analysis by heart failure definition: diagnostic code vs. imaging criteria. AUROC, area under the receiver operating characteristic curve.

Subgroup analysis based on heart failure (HF) definition revealed consistent performance of AI-ECG models across different diagnostic criteria. The diagnostic code-defined group (7 cohorts, *n* = 951,654) demonstrated a pooled AUROC of 0.765 (95% CI: 0.743–0.786), indicating robust discriminative ability (Figure 6). Substantial heterogeneity was observed (*I*^2^ = 89%), attributable to variations in population characteristics, study design, and healthcare settings. The imaging-defined group (LVEF < 50%, 1 cohort, *n* = 231,285) showed comparable performance with an AUROC of 0.810 (0.800–0.820). The complete overlap between the prediction interval of the diagnostic code group (0.692–0.826) and the point estimate of the imaging group suggests no statistically significant difference in model performance between definition types, though this interpretation is limited by the single study in the imaging subgroup.

These findings indicate that AI-ECG models maintain predictive utility regardless of HF definition methodology. The diagnostic code-based approach offers practical advantages for large-scale screening applications, while imaging-based definitions may provide more objective endpoint adjudication. The observed heterogeneity underscores the need for standardized outcome definitions and validation protocols in future multicenter studies. Further research should prioritize inclusion of additional imaging-defined cohorts to strengthen comparative analyses and evaluate definition-specific impacts on model calibration and clinical utility.

## 4 Discussion and conclusion

### 4.1 Principal findings and clinical implications

This systematic review and meta-analysis demonstrates that AI-ECG models possess moderate to good discriminatory ability for predicting heart failure (HF), with a pooled AUROC of 0.76 (95% CI: 0.74–0.78). This performance is comparable to established clinical risk scores such as the FHS-HF model (AUROC 0.742), suggesting that AI-ECG holds promise as a non-invasive and cost-effective tool. Its operational simplicity—leveraging ubiquitously available ECG data without the need for invasive blood tests or complex clinical parameters—positions it as an ideal candidate for rapid point-of-care screening and global cardiovascular risk reduction strategies, particularly in resource-limited areas.

### 4.2 Performance generalizability and subgroup analyses

Critically, the potential of AI-ECG is underscored by its consistent performance across ethnic groups in our subgroup analyses (AUROC range: 0.77–0.79). Notably, while the technology did not demonstrate universal superiority over traditional models, it showed enhanced performance in specific cohorts, such as the large Asian TSGH population. This variability suggests that AI-ECG may offer the greatest incremental value in populations with specific baseline risk profiles or where traditional risk factors are not fully captured. Therefore, AI-ECG should be viewed not as a blanket replacement for established scores, but as a complementary tool with particular utility in certain contexts.

### 4.3 Sources of heterogeneity and methodological challenges

Significant heterogeneity was observed across the included studies (*I*^2^ = 89%), which can be attributed to several methodological factors. First, inconsistent definitions of heart failure outcomes represent a principal source of variability. While the majority of studies relied on ICD codes extracted from electronic health records, others adopted imaging-confirmed criteria such as left ventricular ejection fraction (LVEF). This fundamental discrepancy in endpoint ascertainment not only limits the direct comparability of model performance but also introduces potential misclassification bias, particularly concerning heart failure with preserved ejection fraction (HFpEF).

Additionally, the diversity in ECG data sources and processing methodologies further contributes to the observed heterogeneity. The performance and generalizability of AI-based ECG models are intrinsically tied to the quality and consistency of the input data. In real-world settings, ECG signals are acquired from highly heterogeneous sources, including native digital recordings from standard 12-lead machines, digitized scans of paper printouts, or abbreviated waveforms from wearable monitoring devices. Consequently, disparities in acquisition hardware, sampling frequency, filter settings, and data format may introduce technical confounders that substantially influence model output.

Finally, cohort-specific performance variations underscore this inconsistency. For instance, larger cohorts derived from real-world electronic health record data demonstrated marginally lower AUROC values, potentially attributable to unmeasured confounders or data noise. Notably, the performance in specific subgroups, such as young Black women in one study (20), showed a significant decline. Collectively, these factors highlight the persistent challenges in methodological standardization and the necessity for unified protocols in data acquisition, model validation, and outcome reporting to improve reproducibility.

### 4.4 Limitations and imperatives for future research

Our analysis has several key limitations that chart a course for future work, including the absence of clinical utility assessments, the inherent “black-box” nature of deep learning models (22), and potential publication bias. To address these gaps, an urgent priority is the design and execution of large-scale, prospective, multi-center trials to determine whether AI-ECG-guided risk stratification actually reduces heart failure incidence or improves patient outcomes compared to standard care—a prerequisite for widespread adoption. Beyond establishing efficacy, future efforts must extend into implementation science, focusing on the seamless integration of these algorithms into clinical workflows. This entails developing interoperable systems that embed AI-ECG analysis within electronic health records and provide intuitive decision support to clinicians at the point of care. Furthermore, the sustainable implementation of AI-ECG hinges on demonstrating its long-term value and equity. This mandates rigorous health economic analyses, particularly in low-resource settings where its operational advantages could be most impactful, coupled with proactive measures to ensure fairness and transparency. Independent third-party validation and periodic auditing are indispensable to mitigate performance disparities across ethnic, and socioeconomic groups and to prevent the exacerbation of existing health inequities. Adopting privacy-preserving frameworks, such as federated learning, could further facilitate continuous model refinement across diverse institutions.

### 4.5 Conclusion

In conclusion, AI-ECG models demonstrate moderate-to-good discriminatory ability for predicting heart failure, with performance comparable to traditional risk models. The technology shows particular promise as a non-invasive, cost-effective screening tool, especially in environments where traditional risk factor collection is challenging. Future research should prioritize robust prospective validation, seamless clinical integration, and a steadfast commitment to equitable deployment. Ultimately, the goal is to identify the specific patient populations and clinical scenarios where AI-ECG provides the greatest incremental value, thereby enabling its transition from a research tool to a clinically actionable solution for personalized HF prevention.

## Data Availability

The original contributions presented in the study are included in the article/Supplementary Material, further inquiries can be directed to the corresponding author.
