# Supplementary material for: Artificial intelligence in electrocardiogram-based prediction of heart failure: a systematic review and meta-analysis
Source: Front Cardiovasc Med. 2026 Jan 2;12:1659298. doi: 10.3389/fcvm.2025.1659298 (PMC12808466; doi:10.3389/fcvm.2025.1659298)
Supplement: Supplementary file 1 [file Datasheet1.pdf]

SUPPLEMENTARY METHODS

Formulation of research question using CHARMS (CHecklist for critical Appraisal and data extraction for systematic Reviews of prediction Modelling Studies):

Table S1:CHARMS

**CHARMS key items to guide framing of review, search strategy and study inclusion and exclusion criteria**	**Comments for this systematic review**
Diagnostic prediction model	Prediction model
Intended scope of the review	Model for prediction
Types of Prediction modelling studies	Prediction model development without external validation in independent data, prediction model development with external validation in independent data, external model validation, possibly with model updating
Target population to whom the prediction model applies	Adults in the general population

Outcome to be predicted	Specific future event, diagnosis of heart failure
Time span of prediction	Any time interval
Intended moment of using the model	Models to be used in the community to predict risk of development of heart failure in the future, and inform targeted screening and/or primary prevention

Search Terms and search strategy with full results

Database(s): Med Pumd <March 1,2005 to March 1, 2025>

Search Strategy:

Table S2:

#	**Searches**	**Results**
1	"Artificial Intelligence" OR "Machine Learning" OR "Deep Learning"	41,478
	(heart failure or congestive heart failure or cardiac insufficiency or cardiac failure).ti,ab.	82,479
2	1 AND 2	41,478
3	"Electrocardiogram" OR "ECG"	13106
4	ROC Curve/ or (stratification or discrimination or discriminate or c-statistic or c statistic or Area under the curve or Calibration or Indices or Algorithm or Multivariable).ti,ab.	661,985

5	Prediction" OR "Early Detection"	12673
6	1 AND 2 AND 3 AND 5	17

Inclusion and exclusion criteria pertaining to variables incorporated in models

In this review we were interested in models that could be used with data routinely-collected in the community to inform the individual risk of heart failure. To make the model useful it should not require additional visits for baseline risk stratification.

In this review, we focus on models capable of predicting an individual's risk of heart failure using routine based 12-lead electrocardiograms. To keep the models as simple and practical as possible, sociodemographic variables are limited to age, sex, and race, while all other demographic variables—such as disease status, blood pressure, height, weight, body mass index, lifestyle factors, laboratory tests, and biomarkers—are excluded.

Applying the PICOTS framework to clarify the intended objectives or purposes of predictive model evaluation

Table S3 PICOTS Prediction Model Evaluation

Study	Cohort	HF cases/total patients (%)	Index model(s)	Comparator model(s)	Setting and intended use of the prediction model	Enrolment period (mean f/u in years)
Kaur 2024	Stanford University Medical Center(USA)	59816/326663(18.3%)	5	0	The impact of age, race, ethnicity and gender on the performance of the AI-ECG model for heart failure prediction.	2008 – 2018(6.8)
Akbilgic 2021	ARIC (USA)	803/14613(5.5%)	1	3	The validation criteria indicate that a 10-second 12-lead electrocardiogram alone can predict the risk of heart failure within 10 years with moderately high accuracy.	1987-1989(N/S)
Dhingra 2025	YNHHS(USA)	9645/231285(4.2%)	1	3	AI-ECG can identify populations with an elevated risk of newly developed heart failure.	2014-2023(4.5)

	UKB(UK)	46/42141(0.1%)	1	2	AI-ECG can identify populations with an elevated risk of newly developed heart failure.	2014-2021(3.1)
	ELSA-Brasil(BRA)	31/13454(0.2%)	1	3	AI-ECG can identify populations with an elevated risk of newly developed heart failure.	N/S(4.2)
Lin 2025	TSGH(CHN）	(-)/539934	1	1	The AI-ECG model predicts the occurrence of major adverse cardiovascular events (MACE).	N/S(5)
Butler 2023	ARIC (USA)	803/14613(5.5%)	1	5	Validating the predictive performance of models for heart failure.	1987-1989(N/S)
	MESA（USA）	239/6736(3.5%)	1	5	Validating the predictive performance of models for heart failure.	N/S

AF, atrial fibrillation; SUMC, Stanford University Medical Center; ARIC, Atherosclerosis Risk in Communities; YNHHS, Yale New Haven Health System; UKB, UK Biobank ; ELSA-Brasil, Brazilian Longitudinal Study of Adult Health; TSGH, Tri-Service General Hospital; MESA, Multi-Ethnic Study of Atherosclerosis; ECG, electrocardiogram ; LVEF, left ventricular ejection fraction; SNOMED, Systematized Nomenclature of Medicine; C-EEMRR, clinical events based on expert medical record review; N/S, not specified.

PROBAST+AI: Justifications for assessments for specific signalling questions

Each model was assessed for quality and risk of bias as either "high", "unclear" or "low" in four domains (Participants and data sources, predictors, outcomes and analysis) through a range of signalling questions. Applicability to our review question was assessed for each model in three domains (participants, predictors and outcomes) using the same scale.

Risk of bias

Domain 1: Participants and data sources

Signalling question 3: Did the in- and exclusions of study participants result in a representative dataset?

Our review focuses on the generalizability research of artificial intelligence models based on 12-lead electrocardiograms. Therefore, if the study has already excluded populations with heart failure, this signal issue will be marked as "Yes."

Domain 3: Analysis

Signalling question 4: Was the time interval between predictor assessment and outcome assessment appropriate?

We consider an interval of ≥5 years between evaluation and outcome assessment to be appropriate, and this signal issue will be marked as "Yes."

Domain 4: Analysis

Signalling question 3: Were participants with missing or censored data handled appropriately in the analysis?

We evaluated the risk of bias due to missing data through the following assumptions: If a study completely failed to mention missing data, it was highly likely that missing data existed but was not considered, thus labeled as "N or PN" and judged as "unclear" risk of bias. When any degree of missing data was present but no imputation methods were employed, or no analysis was conducted to assess whether including missing values would affect performance metrics, then this signaling question was labeled as "N or PN," and the entire domain was judged as "unclear" risk of bias.

Table S4: Risk of bias across all included studies

Model	Study	ROB Participants	ROB Predictors	ROB Outcome	ROB Analysis	ROB Overall
Deep learning	Akbilgic 2021(ARIC/undifferentiated)	L	L	L	L	L
Deep learning	Butler 2023(ARIC/undifferentiated)	L	L	U	U	U
Deep learning	Butler 2023(MESA/undifferentiated)	L	L	U	U	U
Deep learning	Dhingra 2025(YNHHS/undifferentiated)	L	L	L	L	L
Deep learning	Dhingra 2025(UKB/undifferentiated)	L	L	L	L	L
Deep learning	Dhingra	L	L	L	L	L

	2025(ELSA-Brasil/undifferentiated)					
Deep learning	Kaur 2024(SUMC/Asian)	L	L	L	U	L
Deep learning	Kaur 2024(SUMC/Hispanic)	L	L	L	U	L
Deep learning	Kaur 2024(SUMC/Non-Hispanic White)	L	L	L	U	L
Deep learning	Kaur 2024(SUMC/Black or African American)	L	L	L	U	L
Deep learning	Lin 2025(TSGH/Asian)	L	L	L	U	L

SUMC, Stanford University Medical Center; ARIC, Atherosclerosis Risk in Communities; YNHHS, Yale New Haven Health System; UKB, UK Biobank ; ELSA-Brasil, Brazilian Longitudinal Study of Adult Health; TSGH, Tri-Service General Hospital; MESA, Multi-Ethnic Study of Atherosclerosis; AUROC, Area Under the Receiver Operating Characteristic Curve.
